# Educational Impact of Artificial Intelligence‐Navigation Surgery on Anatomical Landmark Recognition in Medical Students

**DOI:** 10.1002/ags3.70149

**Published:** 2025-12-15

**Authors:** Shigeo Ninomiya, Yusuke Matsunobu, Tabito Oyama, Hiroomi Takayama, Takashi Masuda, Teijiro Hirashita, Yuichi Endo, Tatsushi Tokuyasu, Masafumi Inomata

**Affiliations:** ^1^ Department of Gastroenterological and Pediatric Surgery Oita University Faculty of Medicine Yufu Japan; ^2^ Department of Healthcare AI Data Science Oita University Faculty of Medicine Yufu Japan; ^3^ Department of Comprehensive Surgery for Community Medicine Oita University Faculty of Medicine Yufu Japan; ^4^ Department of Information Systems and Engineering, Faculty of Information Engineering Fukuoka Institute of Technology Fukuoka Japan

**Keywords:** artificial intelligence, navigation surgery, surgical education

## Abstract

**Background:**

We evaluated the educational impact of artificial intelligence (AI)‐navigation surgery for medical students which provides real‐time anatomical landmark recognition during laparoscopic cholecystectomy (LC).

**Methods:**

Thirty fifth‐year medical students were randomly assigned to three groups: surgeon‐guided (*n* = 10), self‐learning (*n* = 10), and AI‐learning (*n* = 10). Each group annotated anatomical landmarks, extrahepatic bile duct (EHBD), cystic duct (CD), Rouvière's sulcus (RS), the base of liver segment 4 (S4), before and after training. The AI‐learning group received real‐time feedback using a deep learning segmentation model (HyperSeg). Learning outcomes were quantitatively assessed and compared to expert annotations using Dice coefficients, and post‐study questionnaires were analyzed to evaluate understanding of anatomy and surgical procedures.

**Results:**

The mean Dice coefficients in the surgeon‐guided (0.450 ± 0.025) and AI‐learning groups (0.432 ± 0.038) were significantly higher in comparison to the self‐learning group (0.351 ± 0.057, *p* = 0.00006). In an itemized analysis, significant improvements were observed in EHBD and RS recognition, but not in CD or S4 recognition. In the post‐study questionnaire assessing anatomical understanding and the ability to comprehend the surgeon's perspective and intentions, the surgeon‐guided group showed significantly better results in comparison to the self‐learning group (*p* < 0.001 for each comparison). However, there was no significant difference between the AI‐learning and self‐learning groups.

**Conclusions:**

AI has the potential to complement surgeon's guidance, reducing faculty burden while maintaining educational quality in surgical education.

## Graphical abstract

This study evaluated the educational impact of artificial intelligence (AI)‐navigation surgery that provides real‐time anatomical landmark recognition during laparoscopic cholecystectomy for medical students. Thirty students were randomized into surgeon‐guided, self‐learning, and AI‐learning groups, and their performance was assessed using Dice coefficients and questionnaires. The AI‐learning group achieved learning outcomes comparable to surgeon‐guided instruction, suggesting that AI can maintain educational quality while reducing the teaching burden on surgeons.

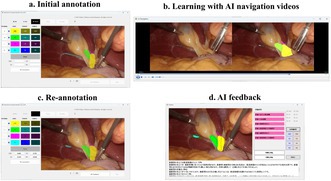

## 1 Introduction

Due to the work style reform for physicians implemented in 2024, surgeons and faculty members now have less time to dedicate to medical education. In fact, some reports suggest that restrictions on physicians' working hours may lead to a decline in the quality of medical education [1, 2]. On the other hand, university physicians in Japan are overwhelmed with clinical duties, leaving them with limited time for research and educational activities [3]. In this era, there is a growing need to establish methods of medical education that are more efficient and less burdensome for faculty members, while maintaining high educational quality.

Our institution was the first in the world to develop an artificial intelligence (AI) navigation surgery system that provides real‐time guidance on anatomical landmarks during laparoscopic cholecystectomy (LC) [4]. In brief, this system identifies key anatomical landmarks, the extrahepatic bile duct (EHBD), the cystic duct (CD), Rouviere's sulcus (RS), and the base of liver segment 4 (S4), and displays them in real time during surgery. We previously reported that this system enhances the ability of both expert and junior surgeons to recognize anatomical landmarks and is also useful for surgical education [5]. However, its effectiveness for medical student education remains to be clarified.

The purpose of this study is to investigate the impact of AI‐navigation surgery on the education of medical students.

## 2 Methods

### 2.1 Quantitative Evaluation of Anatomical Landmark Recognition

The study included 30 fifth‐year medical students. These students were randomly divided into three groups: a surgeon‐guided group (*n* = 10), a self‐learning group (*n* = 10), and an AI‐learning group (*n* = 10). The degree of learning regarding the anatomical landmarks in LC was compared among the three groups. Prior to the experiment, all students attended a lecture on the procedural steps of LC given by a surgeon and viewed intraoperative videos. They then annotated anatomical landmarks using the following protocol. As previously reported, the anatomically important landmarks in LC are EHBD, CD, RS and S4; these serve as useful reference points for the prevention of bile duct injury [4]. All groups performed annotations of these specific landmarks. After each group completed training with 20 cases, they performed annotation on 10 images as a test. The annotations created by a board‐certified gastroenterological surgeon of the Japan Society for Endoscopic Surgery (Y.E.) were used as the **ground truth**, representing the reference standard for each anatomical landmark. The concordance between student annotations and the ground truth was quantitatively assessed using Dice coefficients. The data analysis was performed only after all 30 participants had completed the experiment. For each group, we evaluated the overall annotation concordance rate together with the concordance rates for EHBD, CD, RS, and S4 [5].

**FIGURE 1:**
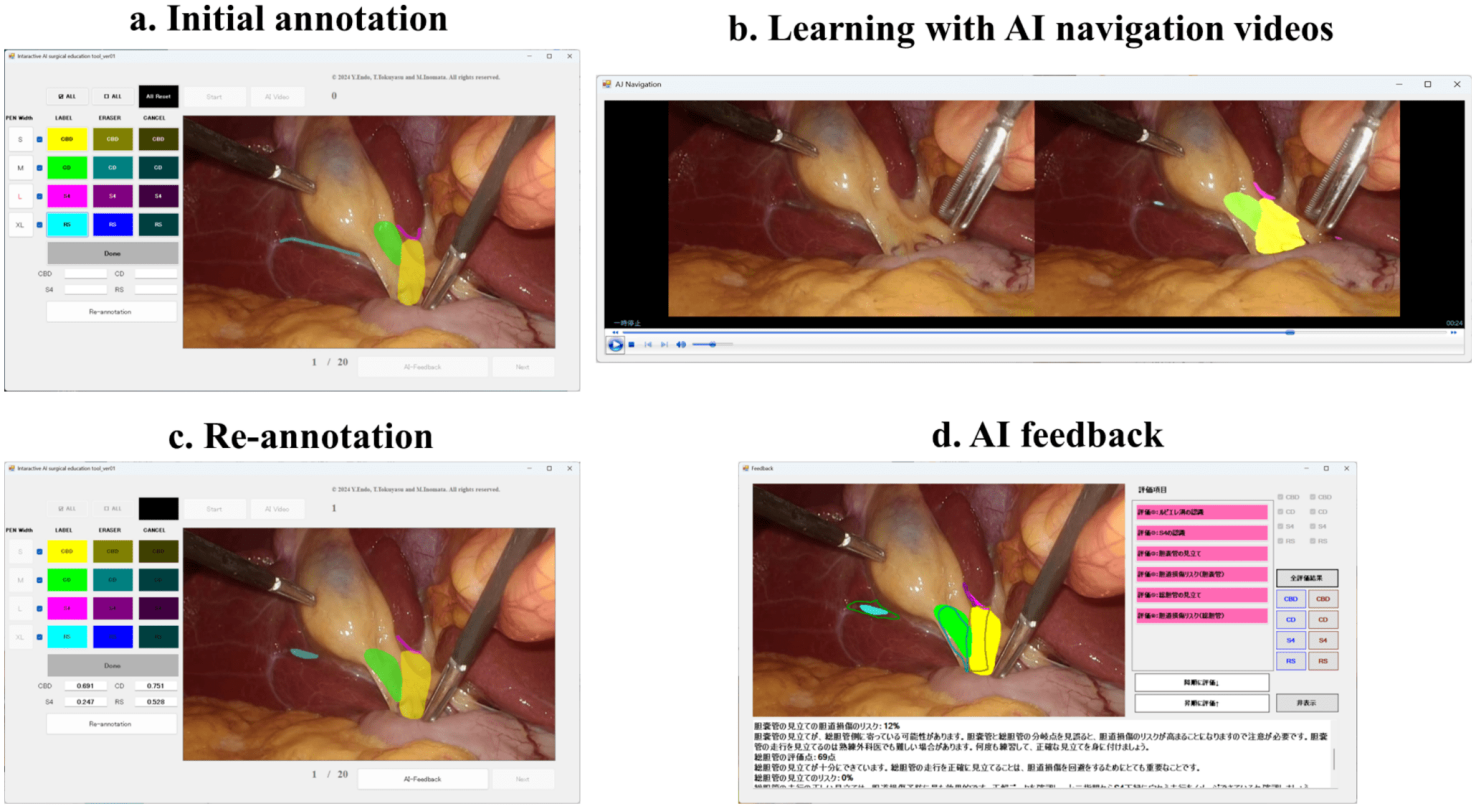
Educational framework of the AI‐learning group. (a) Initial annotation of anatomical landmarks, (b) learning with AI navigation videos, (c) re‐annotation, and (d) AI‐generated feedback using Dice coefficients.

- **Surgeon‐guided group (*n* = 10):** Students annotated anatomical landmarks from 20 LC cases under the same surgeon's supervision (S.N.) to ensure consistent guidance. They then watched a 30‐s video and re‐annotated the landmarks. Subsequently, they independently annotated 10 additional cases without surgeon guidance as a test.
- **Self‐learning group (*n* = 10):** Students independently annotated anatomical landmarks on 20 LC images. They then watched a 30‐s surgical video (without AI assistance) and re‐annotated the landmarks. Afterwards, they independently annotated 10 additional cases as a test.
- **AI‐learning group (*n* = 10):** Students annotated anatomical landmarks on 20 LC images (Figure 1a). They then watched videos incorporating the AI‐assisted surgical guidance and re‐annotated the landmarks (Figure 1b,c). Subsequently, the medical students regarded the agreement between their own answers and the ground truth as AI feedback, and this concordance was used to evaluate the effectiveness of their learning (Figure 1d). This educational framework was designed to facilitate visual learning of the spatial relationships among anatomical structures during dissection of Calot's triangle. Subsequently, they independently annotated 10 additional cases as a test.

### 2.2 Post‐Study Questionnaire

**FIGURE 2:**
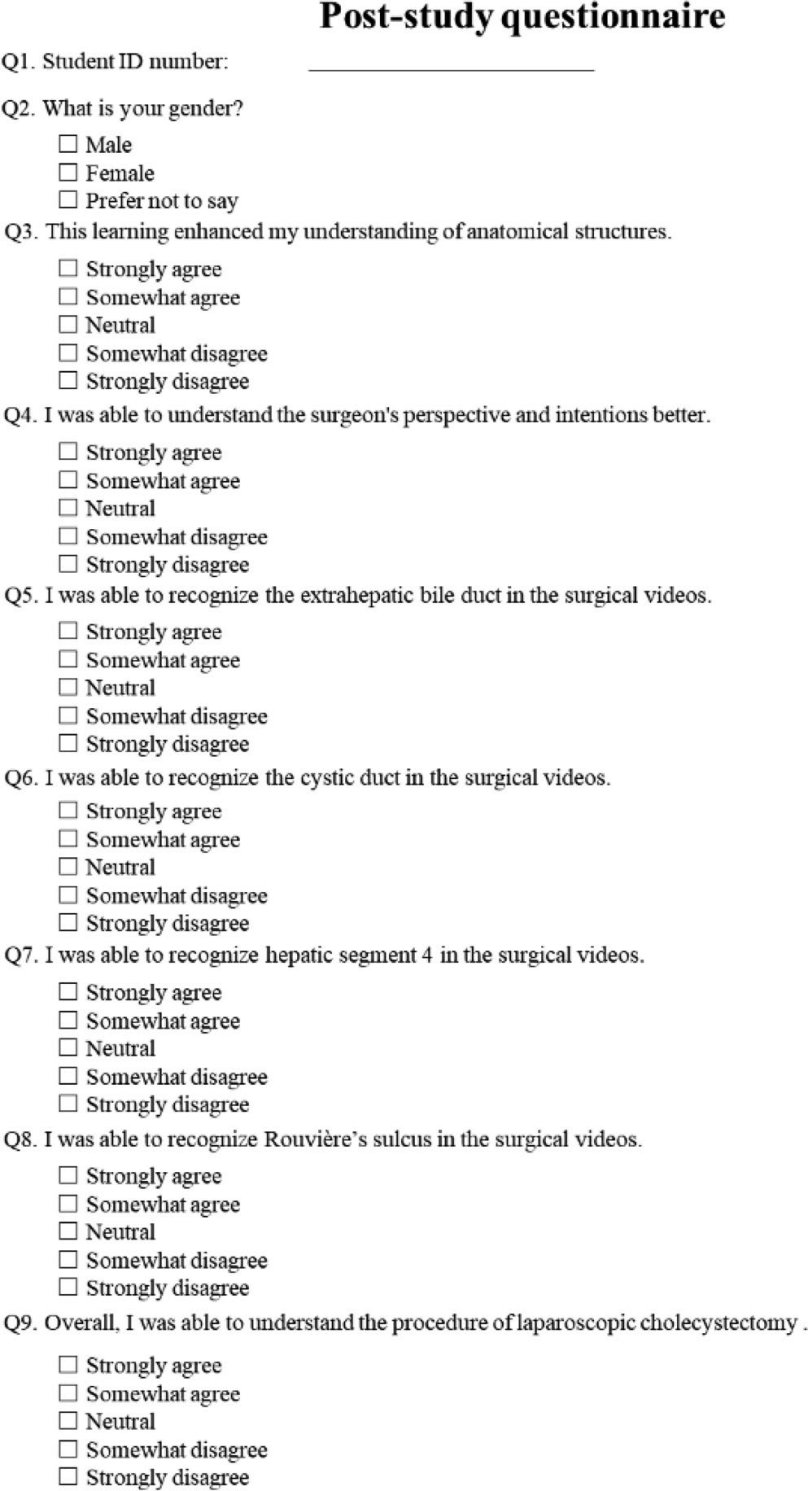
Post‐study questionnaire administered to participating medical students. The questionnaire consisted of nine items assessing the understanding of anatomical structures and surgical procedures.

After the study, a questionnaire was administered to all participating medical students. The questionnaire was conducted using Google Forms, requiring only the student ID number, and responses were collected anonymously. The questionnaire, as shown in Figure 2, consisted of 9 items and primarily assessed the understanding of anatomical structures and surgical procedures.

### 2.3 Development of the AI Model

We developed and utilized a deep learning‐based segmentation AI model, HyperSeg, to assist in the recognition of critical anatomical landmarks during LC. HyperSeg is capable of providing highly accurate, real‐time segmentation of surgical images and was also applied in our previous study that utilized AI‐based landmark teaching during laparoscopic gastrectomy [6].

### 2.4 Training Data and Annotation

A total of 74 LC videos purchased from Surg Storage Inc., and performed at various medical institutions were reviewed. We excluded cases with acute or chronic cholecystitis to ensure clear visualization of anatomical landmark. From these videos, scenes depicting the dissection of Calot's triangle were extracted, and 1960 still images were sampled at a rate of 1 frame per second. Images in which the anatomical landmarks were clearly visible were subsequently selected for annotation. All selected images were manually annotated by board‐certified gastroenterological surgeons certified by the Japanese Society of Gastroenterological Surgery. The target anatomical landmarks and the number of annotated images were as follows: EHBD (1892 images); CD (1771 images); S4 (1851 images); RS (724 images).

### 2.5 Statistical Analysis

For quantitative analysis, the Dice coefficients of the 10 images in each group were averaged and analyzed by one‐way analysis of variance (ANOVA). The assumptions of normality and homogeneity of variances were confirmed using the Shapiro–Wilk and Levene's tests, respectively. As all three groups were independent, equally sized, and satisfied these assumptions, ANOVA followed by Tukey's Honestly Significant Difference test was applied. For the questionnaire results, the Kruskal‐Wallis test was performed to examine whether there were significant differences in the median values among the groups, and Dunn's test was conducted to identify which group pairs showed significant differences using responses measured on a Likert scale. The results of the quantitative analysis were presented as the mean ± standard deviation (SD), and the results of the questionnaire values were presented as the median ± SD. *p* values of < 0.05 were considered to indicate statistical significance.

## 3 Results

### 3.1 Quantitative Evaluation of Anatomical Landmark Recognition

**FIGURE 3:**
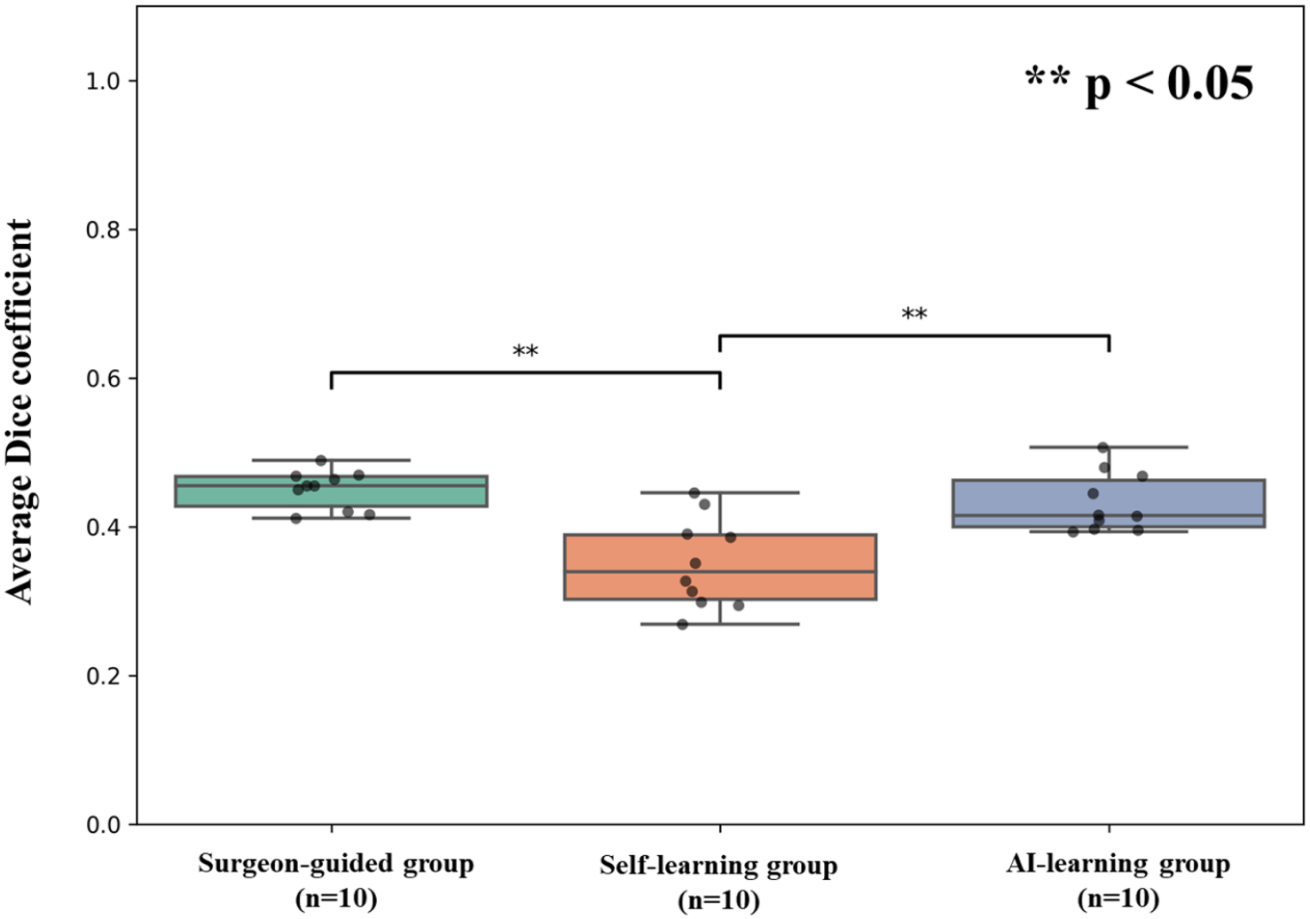
Average Dice coefficients of the three groups. The surgeon‐guided and AI‐learning groups achieved significantly higher Dice coefficients in comparison to the self‐learning group (**p < 0.05).

**FIGURE 4:**
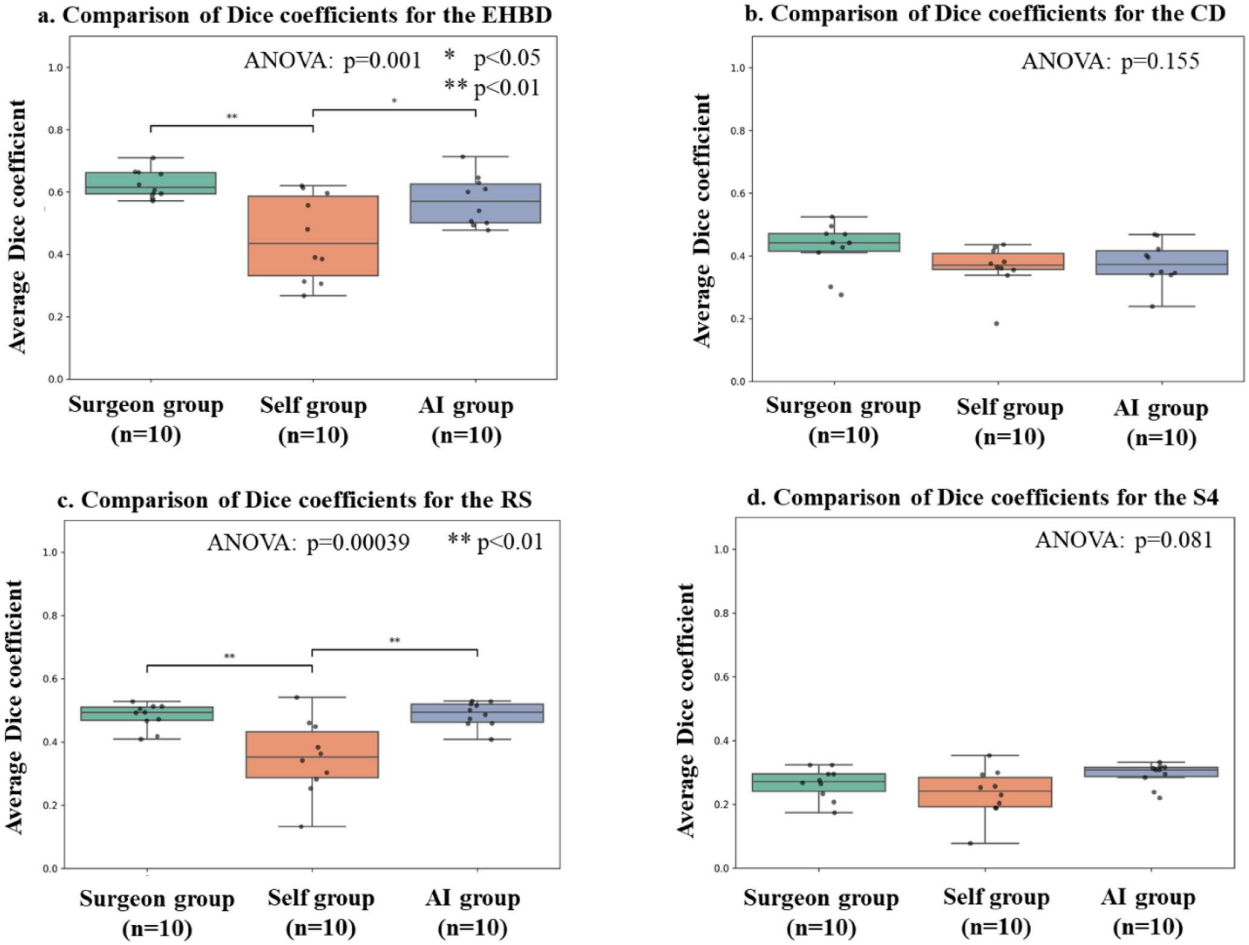
Itemized analysis of Dice coefficients for each anatomical landmark. (a) Extrahepatic bile duct (EHBD), (b) cystic duct (CD), (c) Rouvière's sulcus (RS), and (d) liver segment 4 (S4). Significant differences were observed for EHBD and RS, but not for CD and S4. ANOVA, one‐way analysis of variance.

The results of the quantitative analysis of anatomical landmark recognition are shown in Table 1. The overall Dice coefficients for the surgeon‐guided, self‐learning, and AI‐learning groups were 0.450 ± 0.025, 0.351 ± 0.057, and 0.432 ± 0.038, respectively. The surgeon‐guided and AI‐learning groups showed significantly better learning outcomes in comparison to the self‐learning group (*p* = 0.00006) (Figure 3). In the subgroup analysis, both the surgeon‐guided and AI‐learning groups showed significantly better Dice coefficients in comparison to the self‐learning group (*p* < 0.05 for each). In contrast, no significant difference was observed between the surgeon‐guided and AI‐learning groups (*p* = 0.65). In the itemized analysis, significant differences were observed for EHBD and RS, whereas no significant differences in Dice coefficients were found for CD and S4 (Figure 4).

**TABLE 1:** Dice coefficient comparing the average test image of each group with the model answer (ground truth).

	Surgeon‐guided group	Self‐learning group	AI‐learning group	*p*
Overall	0.450 ± 0.025	0.351 ± 0.057	0.432 ± 0.038	0.00006
EHBD	0.627 ± 0.043	0.453 ± 0.130	0.572 ± 0.075	0.001
CD	0.426 ± 0.076	0.364 ± 0.067	0.377 ± 0.065	0.155
RS	0.481 ± 0.038	0.351 ± 0.111	0.488 ± 0.037	0.00039
S4	0.266 ± 0.046	0.235 ± 0.072	0.294 ± 0.035	0.081

*Note:* Values are presented as the mean ± standard deviation. Abbreviations: CD, cystic duct; EHBD, extrahepatic bile duct; RS, Rouviere's sulcus; S4, the base of liver segment 4.

### 3.2 Post‐Study Questionnaire

**FIGURE 5:**
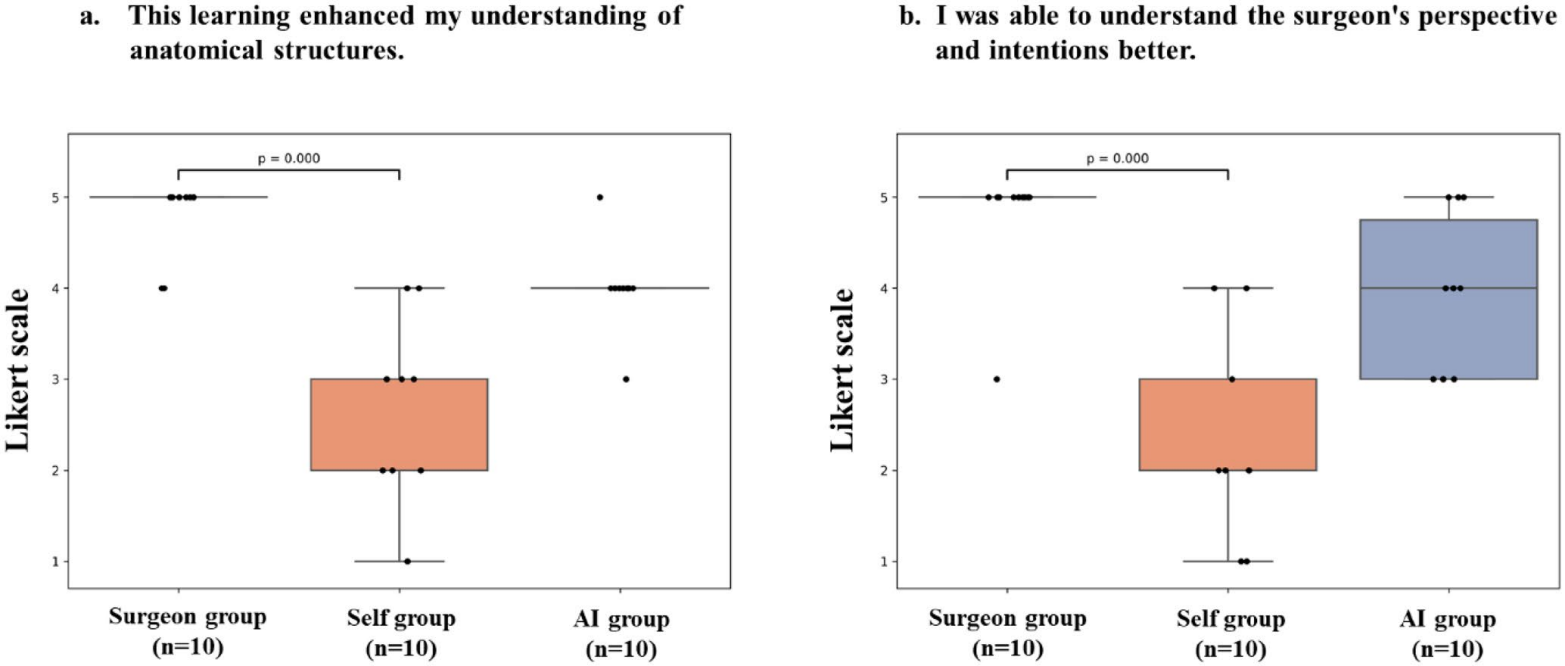
Post‐study questionnaire results on understanding of anatomy and surgical perspective. (a) “This learning enhanced my understanding of anatomical structures.” (b) “I was able to understand the surgeon's perspective and intentions better.” Likert scale analysis across the three groups.

**FIGURE 6:**
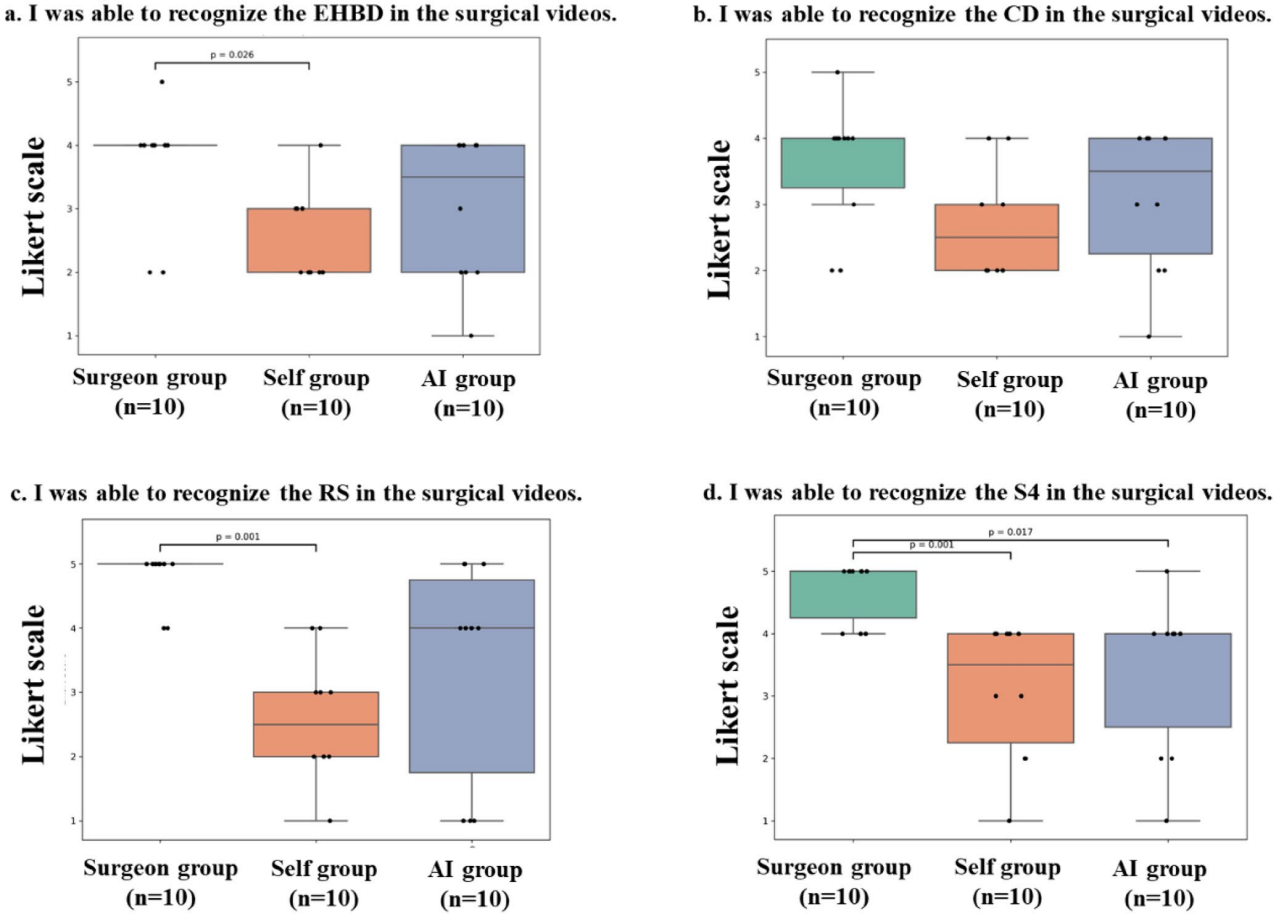
Post‐study questionnaire results on anatomical landmark recognition. (a) Extrahepatic bile duct (EHBD), (b) cystic duct (CD), (c) Rouvière's sulcus (RS), and (d) liver segment 4 (S4). Results of the Likert scale analysis.

**FIGURE 7:**
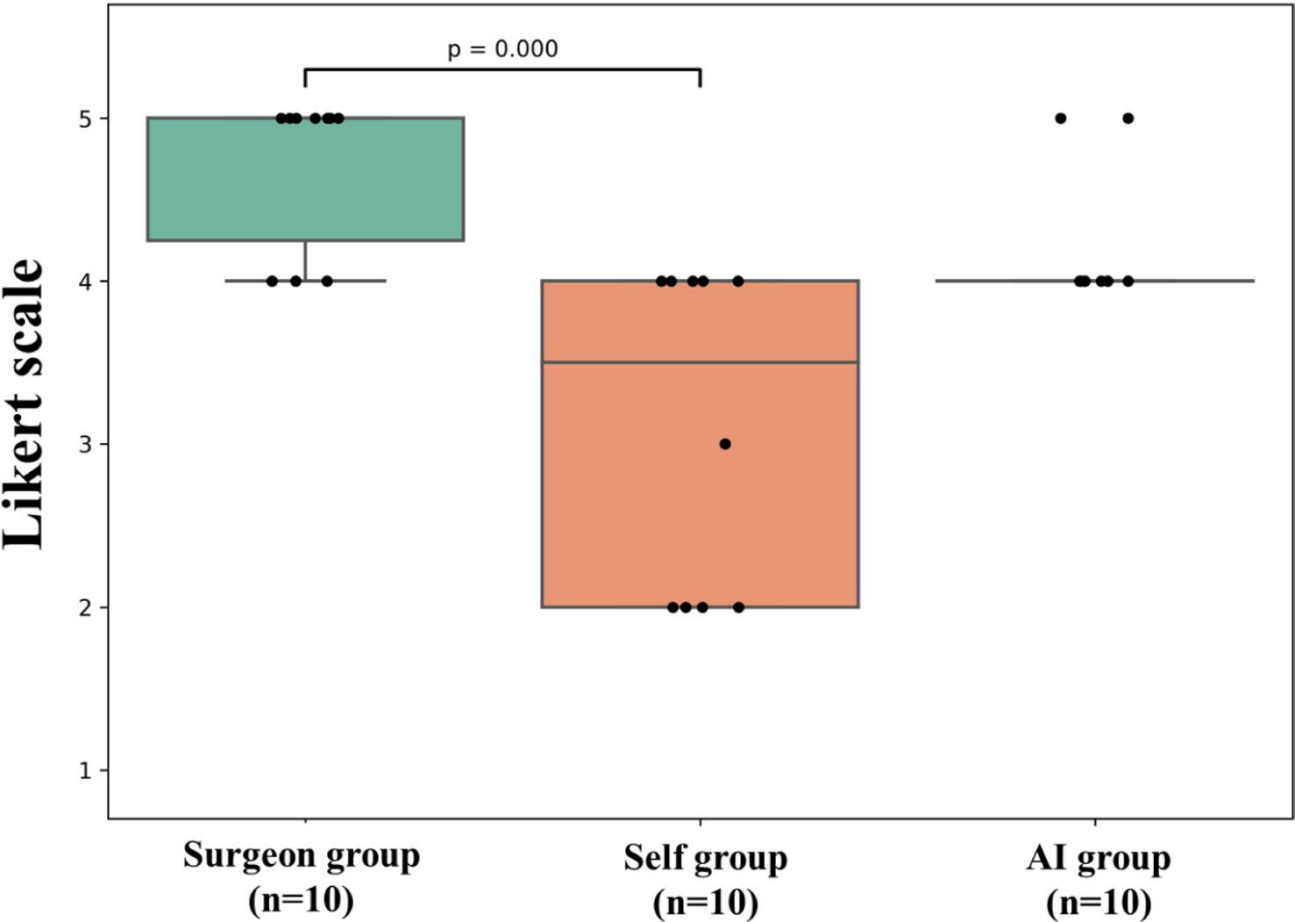
Post‐study questionnaire results on overall understanding of laparoscopic cholecystectomy. Responses of the surgeon‐guided, self‐learning, and AI‐learning groups.

The results of the analysis of the seven questionnaire items (Q3–9) are shown in Table 2. For the items, ‘This learning enhanced my understanding of anatomical structures’ and *‘*I was able to understand the surgeon's perspective and intentions better*’*, the surgeon‐guided group showed significantly better results in comparison to the self‐learning group. Specifically, for the first item, the median score in the surgeon‐guided group was 4.8 ± 0.4, while that in the self‐learning group was 2.7 ± 1.0 (*p* < 0.001). For the second item, the median score in the surgeon‐guided group was 4.8 ± 0.6, while that in the self‐learning group was 2.3 ± 1.1 (*p* < 0.001). However, the median scores of the AI‐learning and self‐learning groups did not differ to a statistically significant extent (Figure 5). In the itemized analysis of anatomical landmark recognition assessed by the post‐study questionnaire (Figure 6, Table 2), the surgeon‐guided group achieved significantly higher scores for the recognition of the EHBD, RS, and S4 in comparison to the self‐learning group. However, no significant differences in these items were observed between the AI‐learning and self‐learning groups. Furthermore, regarding the overall understanding of LC (Figure 7, Table 2), the surgeon‐guided group also showed significantly better results than the self‐learning group. In contrast, the AI‐learning group did not demonstrate a significant improvement in comparison to the self‐learning group.

**TABLE 2:** Post‐study questionnaire results.

	Surgeon‐guided group	Self‐learning group	AI‐learning group	*p*
Q3	4.8 ± 0.4	2.7 ± 1.0	4.0 ± 0.5	*p* < 0.001*
Q4	4.8 ± 0.6	2.3 ± 1.1	3.9 ± 0.9	*p* < 0.001*
Q5	3.7 ± 0.9	2.5 ± 0.7	3.0 ± 1.2	*p* < 0.05*
Q6	3.6 ± 1.0	2.7 ± 0.8	3.1 ± 1.1	n.s.*, n.s.**
Q7	4.7 ± 0.5	3.1 ± 1.1	3.4 ± 1.3	*p* < 0.01*, *p* < 0.05**
Q8	4.8 ± 0.4	2.6 ± 1.0	3.4 ± 1.7	*p* < 0.05*
Q9	4.7 ± 0.5	3.1 ± 1	4.2 ± 0.4	*p* < 0.001*

*Note:* Values are presented as the median ± standard deviation (SD).

* Comparison between the Surgeon‐guided group and the Self‐learning group. n.s., not significant.

** Comparison between the Surgeon‐guided group and the AI‐learning group.

## 4 Discussion

The recent advancements in AI have been remarkable, and ChatGPT, a leading example of generative AI, gained 100 million users within just two months of its release in 2022 [7]. In medical education, AI has been also increasingly utilized across various fields. McCarrick et al. reported that history‐taking training using ChatGPT improved students' history‐taking skills and enhanced their confidence in conducting interviews [8]. Rao et al. also reported the usefulness of a large language model‐based tool for surgical oral examinations [9]. While there are reports on the usefulness of text‐generating AI tools such as ChatGPT in medical education, there are few reports demonstrating the educational effectiveness of AI, particularly AI‐assisted navigation surgery. Kinoshita et al. reported, based on a questionnaire survey, that the use of AI in medical education improved nerve recognition during rectal cancer surgery [10]. Similarly, Tomioka et al. reported that AI was useful for student education in laparoscopic hepatectomy [11]. Khan et al. reported that the use of AI in pituitary tumor surgery improved anatomical recognition among medical students [12]. However, in all of these studies, students participated as part of ancillary investigations conducted to validate newly developed AI systems. In contrast, the present study is the first to focus on whether an existing AI system is useful for medical student education.

One of the key advantages of AI is its ability to process and evaluate large and complex datasets beyond human capabilities [13]. In contrast, humans often rely on heuristic biases when making judgments, which may be inaccurate or subjective. In a randomized clinical trial by Fazlollahi et al., an AI‐based tutoring system demonstrated superior effectiveness relative to remote expert instruction in teaching simulated surgical skills to medical students [14]. Yilmaz et al. reported that a group trained with real‐time AI feedback achieved better learning outcomes than a group instructed by in‐person experts [15]. In our study, the outcomes of the surgeon‐guided and AI‐learning groups were comparable. Although the superiority of AI was not demonstrated, its use for instruction remains meaningful, as it has the potential to reduce the burden on surgeons. Furthermore, another advantage of AI is that it allows repeated learning without direct supervision from surgeons. In this study, the number of learning cases was limited to 20 per group to maintain consistent conditions; however, repeated learning using AI may further enhance its educational superiority.

The itemized analysis revealed significant educational differences in EHBD and RS, whereas no differences were observed in CD and S4. CD is usually covered by fatty tissue, which reduces its visibility, and various anatomical variation are often present; therefore, the educational effect was likely limited. In addition, as shown in Table 1, S4 had a lower Dice coefficient compared with other organs, suggesting that students' understanding of this structure was inherently limited. These factors may account for the absence of differences in CD and S4. Because the educational effect may vary depending on the anatomical characteristics of the organs indicated by AI, further studies are needed to identify which organs and surgical procedures are most suitable for AI‐based education.

AI can provide education uniformly and without subjective bias. Although quantitative evaluation was not possible in this study, as shown in the Figure 4, the self‐learning group exhibited a larger standard deviation in comparison to the other groups. This suggests that there was variability in the learning outcomes among the medical students in this group. In contrast, both the surgeon‐guided and AI‐learning groups showed smaller standard deviations than the self‐learning group, indicating that both approaches enabled more consistent learning.

This study highlighted challenges associated with implementing AI in medical education. Although quantitative evaluation showed that AI learning had educational effects comparable to those of surgeon‐guidance, the post‐study questionnaire did not show a significant difference between the AI‐guided and self‐learning groups in responses to items such as “This learning enhanced my understanding of anatomical structures” and “I was able to understand the surgeon's perspective and intentions better”. This may be attributed to the fact that AI, providing unidirectional instruction, makes interactive, bidirectional learning difficult. In patient interviews, interactive training using large language models has been reported to be useful [8]; thus, future development of bidirectional surgical education systems will be necessary.

This study had several limitations. First, this study involved a small sample size, with a total of 30 medical students (10 in each group). However, this number was considered adequate based on previous educational studies with similar designs [10, 11, 12]. Second, due to the small sample size, we were unable to statistically verify whether the use of AI shortened the learning time. Third, it was not possible to standardize the students' prior knowledge or abilities. In addition, due to the nature of the system used in this study, double blinding was not feasible. Finally, the study employed a unique annotation technique, and whether it truly reflects educational effectiveness remains open to discussion.

## 5 Conclusions

In conclusion, AI navigation surgery provides an educational effect comparable to that of surgeons, reducing surgeons burden while maintaining educational quality. However, the establishment of interactive, bidirectional instruction is required.

## Author Contributions

**Shigeo Ninomiya:** conceptualization, methodology, investigation, writing – original draft, data curation. **Yusuke Matsunobu:** conceptualization, methodology, investigation, writing – review and editing. **Tabito Oyama:** investigation, data curation, writing – review and editing, conceptualization. **Hiroomi Takayama:** software. **Takashi Masuda:** software. **Teijiro Hirashita:** software. **Yuichi Endo:** validation. **Tatsushi Tokuyasu:** software, supervision. **Masafumi Inomata:** supervision, writing – review and editing.

## Funding

The authors have nothing to report.

## Ethics Statement

This study was approved by the ethic committee of Oita University (Approval No. 3153‐D71). Written informed consent was obtained from all medical students who participated in the study.

## Conflicts of Interest

Masafumi Inomata has financial conflicts of interest (Olympus Co. Ltd., SB KAWASUMI Co. Ltd., and Aderance Co. Ltd.). In addition, he serves on the Editorial Board of this journal. The other authors declare no conflicts of interest in association with the present study.

## Data Availability

All data generated or analyzed during this study are included in this published article.
